# The Nemesis of Specialism

**Published:** 1899-04-01

**Authors:** 


### The Nemesis of Specialism.

One of the marked features of the medicine of to-day is the tendency exhibited by its professors to devote their energies to certain limited departments of practice. Many there are no doubt who, even while so doing, feel a certain regret at such a limitation of their field of work, and take no pride in the fact that by force of circumstances they find themselves arrayed among the army of specialists. Others, however, glory in it, and maintain that all advance in medicine is, and must be, the outcome of its division into special departments, and in this they are thoroughly backed by public opinion. Heart, lungs, liver; eyes, nose, ears; uterine ailments, sexual ailments, mental ailments; operative surgery in every possible form —each operator being specially known and run after for some particular operation; the ordering of massage, the prescription of baths; gout, skin diseases, the removal of superfluous hair; down almost to chiropody, professional work is chopped up into little sections, each with its little group of "experts," who, in regard to their own minute specialties, are immensely wise, but take to themselves no shame in confessing foolishness concerning everything else.

So far so good; all is within the profession, and we must not complain. But this sort of thing brings its own Nemesis. It is impossible to go on narrowing down the field of the various specialties to the extent that is now being done without tempting the great public to whom we bow so readily, and who, in truth, have in their pockets the fees on which we live, to ask, Can it really be necessary that the man who practises one of these minute specialties should not only have been taught the anatomy and physiology of the whole of the human frame, but should have obtained a complete legal qualification in medicine, in surgery, and in obstetrics? That a fallacy underlies such a suggestion is, of course, obvious, and everyone who knows the complexity of the human frame and the interdependence of its various parts, recognises at once that no one should be allowed to meddle with it who does not know all about it. But to the public this is not so plain, and one of the little pin pricks with which the medical profession is now being punished for having chopped up the medical art into such little fragments is that unqualified persons are picking up these fragmentary specialties, and are using them to their own advantage. Midwives, masseurs, medical electricians, bath men, removers of superfluous hair, spectaclemakers, and a crowd of others are all becoming very knowing people indeed on their own subjects, and some of them are by no means backward in making parade of various diplomas or certificates which, while literally only certifying t[illegible] knowledge or to training, are, it is to be feared, to[illegible] often accepted by the public as indicating thei[illegible] capacity if not their right to treat disease.

The thin end of the wedge was inserted whe[illegible] the dentists were admitted to a separate registe[illegible] The principle was then accepted that in the trea[illegible]ment of diseases of a certain part of the frame [illegible] less complete knowledge of the whole body and o[illegible] general diseases was admissible than was adequat[illegible] in the case of the general surgeon or physician[illegible] No doubt the dentist makes up for the incomplete[illegible]ness of his knowledge by an extremely perfec[illegible] acquaintance with every detail pertaining to hi[illegible] own department. But then that is just what th[illegible] other people say about themselves. Each of them maintains that, as the British workman would say, "he knows his own job," and the principle having once been admitted that the specialist need not a[illegible] the same time be wise all round, it is difficult t[illegible] see where the mischief is to end. Already th[illegible] midwives are clamouring for a Register, or a Rol[illegible] as they now call it, and for a legal recognition o[illegible] their right to practice; the spectaclemakers ar[illegible] already granting a diploma which, although, o[illegible] course, not giving any right to treat disease, i[illegible] undoubtedly accepted by the public as a certificat[illegible] of capacity to treat certain conditions of the ey[illegible] which it would be hard to differentiate from di[illegible]eases; while it is notorious that many sanitar[illegible] inspectors, who are now anxious to become a close corporation, look upon themselves as quite as good in questions of public health as the medical officers whose servants they ought to be. Where the matter is to end we confess we do not see, more especially in view of the fact, which we fear is true, that these spectaclemakers do know more about errors of accommodation, that these bathmen do know more about the bath treatment of gout and rheumatism, and that these electrolytic hair removers do know more about the treatment of hypertrichiasis than the average medical practitioner. That is where the difficulty comes in. Looking upon the whole question from the professional point of view, we can have no doubt that none of these interlopers should be allowed for a moment, and that no recognition should be given to the treatment of disease by any who are not on the Medical Register. Unfortunately, the professional view is not everything, and people are all too ready to raise the cry of trades unionism when they see any attempt made to prevent a man "who knows his job" from exercising his ingenuity upon all who choose to submit themselves to it, and unfortunately the thin end of the wedge has already been admitted.

